# The role of conversation in health care interventions: enabling sensemaking and learning

**DOI:** 10.1186/1748-5908-4-15

**Published:** 2009-03-13

**Authors:** Michelle E Jordan, Holly J Lanham, Benjamin F Crabtree, Paul A Nutting, William L Miller, Kurt C Stange, Reuben R McDaniel

**Affiliations:** 1 Department of Educational Psychology, College of Education, The University of Texas at Austin, Austin, Texas, USA; 2 Department of Information, Risk and Operations Management, McCombs School of Business, The University of Texas at Austin, Austin, Texas, USA; 3 Department of Family Medicine, Robert Wood Johnson Medical School, University of Medicine and Dentistry of New Jersey, New Brunswick, New Jersey, USA; 4 Department of Family Medicine, Center for Research Strategies, University of Colorado Health Sciences Center, Denver, Colorado, USA; 5 Department of Family Medicine, Lehigh Valley Hospital and Health Network, Allentown, Pennsylvania, USA; 6 Departments of Family Medicine, Epidemiology and Biostatics and Sociology, Case Comprehensive Cancer Center, Case Western Reserve University, Cleveland, Ohio, USA

## Abstract

**Background:**

Those attempting to implement changes in health care settings often find that intervention efforts do not progress as expected. Unexpected outcomes are often attributed to variation and/or error in implementation processes. We argue that some unanticipated variation in intervention outcomes arises because unexpected conversations emerge during intervention attempts. The purpose of this paper is to discuss the role of conversation in shaping interventions and to explain why conversation is important in intervention efforts in health care organizations. We draw on literature from sociolinguistics and complex adaptive systems theory to create an interpretive framework and develop our theory. We use insights from a fourteen-year program of research, including both descriptive and intervention studies undertaken to understand and assist primary care practices in making sustainable changes. We enfold these literatures and these insights to articulate a common failure of overlooking the role of conversation in intervention success, and to develop a theoretical argument for the importance of paying attention to the role of conversation in health care interventions.

**Discussion:**

Conversation between organizational members plays an important role in the success of interventions aimed at improving health care delivery. Conversation can facilitate intervention success because interventions often rely on new sensemaking and learning, and these are accomplished through conversation. Conversely, conversation can block the success of an intervention by inhibiting sensemaking and learning. Furthermore, the existing relationship contexts of an organization can influence these conversational possibilities. We argue that the likelihood of intervention success will increase if the role of conversation is considered in the intervention process.

**Summary:**

The generation of productive conversation should be considered as one of the foundations of intervention efforts. We suggest that intervention facilitators consider the following actions as strategies for reducing the barriers that conversation can present and for using conversation to leverage improvement change: evaluate existing conversation and relationship systems, look for and leverage unexpected conversation, create time and space where conversation can unfold, use conversation to help people manage uncertainty, use conversation to help reorganize relationships, and build social interaction competence.

## Background

Those attempting to implement qualitative changes in health care settings often find that intervention efforts progress in surprising ways and outcomes of interventions are not what was expected. Because health care organizations are often viewed as machines, unexpected results are frequently interpreted as resulting from variation and error in intervention processes [1-4]. When health care organizations are viewed as complex adaptive systems our attention is called to relationships [5-9] and thereby to conversations [10]. We then recognize that conversation may be a cause of variation in intervention outcomes. It is our contention that the likelihood of intervention success will increase if the role of conversation is considered in intervention design and implementation regardless of the nature or scope of the intervention.

The purpose of this paper is to present a theory about how conversation influences intervention success in health care organizations. Organizational researchers recognize communication as an important aspect of organizational change processes [11]. Most communication approaches to intervention attempts privilege top-down processes and underemphasize the informal, bottom-up processes that take place during intervention attempts[12]. We specifically examine locally-occurring, informal conversation as one aspect of communication and discuss conversation's role in improving and inhibiting the sensemaking and learning required for successful interventions in health care organizations. We define an intervention broadly as the change strategy itself (e.g., a diabetes registry, treatment guidelines, use of preventive care reminders) and also the way in which the change strategy is implemented (e.g., outside mandates, facilitators, extrinsic motivators). We consider conversation to be a collaborative process in which meaning and organization are jointly created. Conversational participants interact through linguistic exchange improvised in real time [13-16]. Conversation usually takes place through face-to-face interaction, but it may also occur in written mediums, as when conversation is mediated by technology such as in virtual on-line discussions. We limit our discussion of conversation to the informal/unplanned/spontaneous/impromptu talk that occurs as organizational members go about their daily work. Such conversation can take place in formal groupings of people such as during team meetings, as well as in informal situations such as occur in the break room or around the water cooler. We are not referring to planned communication built into the design of an intervention, nor are we referring to the regular conversations necessary to maintain organizational functioning.

To develop our theory we use ideas from sociolinguistics to illustrate useful aspects of conversation in general, and to understand how conversation affects interventions in health care organizations. We use concepts from complex adaptive systems theory to examine the role of conversation in health care interventions. We use these two perspectives to argue that paying attention to conversation can increase the success of change efforts by enhancing sensemaking and learning. In addition to using these two theoretical frames, we draw on our fourteen-year program of research designed to understand and assist primary care practices initiate and sustain improvement changes. This program of research included both descriptive and intervention studies, as noted in Table 1. Throughout this paper we show how conversation affected the outcomes of our own intervention efforts, sometimes blocking, sometimes distorting, and sometimes enhancing them.

**Table 1:** A fourteen-year federally-funded program of research to understand primary care practice change

Project Name (Acronym)	Project funding Source and Dates	Project Description
Direct Observation of Primary Care (**DOPC**)	NCI R01 CA60862 (PI, Stange) 1994–1997	Cross-sectional descriptive study of 4454 patient visits to 138 physicians from 84 practices in Ohio using surveys, chart audits and direct observation of visits

Prevention and Competing Demands in Primary Care (**P & CD**)	AHRQ R01 HS08776 (PI, Crabtree) 1996–1999	Ethnographic comparative case studies of 18 practices in Nebraska using participant observation and depth and key informant interviews

Study To Enhance Prevention by Understanding Practice (**STEP-UP**)	NCI 2R01 CA60862 (PI, Stange) 1999–2000	Group randomized intervention trial of 80 Ohio practices using a facilitator to help practices select and tailor strategies from a cancer prevention toolkit.

Insights from Multimethod Practice Assessment of Change over Time (**IMPACT**)	NCI 3R01 CA60862 (PI, Stange) 2001–2004	Secondary data of STEP-UP to understand why some practices made substantial changes and others none, and to create a theoretical change model.

Using Learning Teams for Reflective Adaptation (**ULTRA**)	NHLBI R01 HL70800 (PI, Crabtree) 2002–2008	Group randomized intervention trial of 60 NJ and PA practices using the IMPACT model and a facilitated a "Reflective Adaptive Process" (RAP) to enhance relationships and cardiovascular disease care.

Our current inquiry began when we turned our attention to an assortment of puzzling events across the five studies noted in Table 1. Examining and comparing studies in an attempt to interpret widely varying outcomes within and across interventions and unanticipated responses of clinics to our interventions, we began to notice some similarities among events and to recognize occasions when conversation qualitatively changed the affect of an intervention. The theory development reported in this paper was informed through examination of a large set of cases. The three stories below help illustrate the kinds of events we observed (practice names are pseudonyms). These stories are representative of others in our dataset, and we refer to these stories throughout the paper to support our discussion. These examples all come from our Using Learning Teams for Reflective Adaptation (ULTRA) study, in which our intervention included a reflective-adaptive process (RAP). In the ULTRA study, cross-functional RAP teams were formed and met weekly with an outside facilitator to identify priority improvement opportunities, discuss potential solutions, and pilot clinical changes [17]. One reason for limiting the three examples to one intervention is that they demonstrate that the variance in the ways the interventions played out was not due to the fact that they came from different studies. It was clear to us that some of the variance in intervention outcomes could be attributed to the unplanned conversations that took place among clinic members.

### Belton Clinic

Belton Clinic, owned by a large hospital network, was a small two-physician practice in a suburban setting, which, on the surface, appeared to be doing well. The physicians and office manager initially seemed excited to be part of the ULTRA study and were hopeful the RAP meetings would improve some "small interpersonal problems." We were also optimistic about how the RAP process would enhance the relationships among clinic members. After only the first few RAP meetings, the intervention hit a stone wall. Belligerent conversations were breaking out everywhere in the clinic and in the RAP meetings. Dr. Roberts began complaining aloud about staff issues and inconsistent and unhelpful meetings with her/his partner, Dr. Smith. The office manager created disruptive conversations throughout the practice including arguing with the RAP facilitator and frequently deflecting practice problems to the hospital network. Dr. Smith said the RAP meetings detracted from generating revenue and weren't productive and then complained that s/he worked harder than everyone else. Staff began talking more about all of their problems but not at the RAP meetings out of fear of potential repercussions from the physicians. Within weeks, the RAP sessions were abandoned and the doctors ceased talking with each other.

### Stanton Family Medicine

Stanton Family Medicine was a three-physician practice with a receptionist and a medical assistant. Just prior to beginning the ULTRA study, they purchased a pediatric practice about ten minutes away, but decided to do ULTRA only at the Stanton site. Prior to the first RAP meeting, a new office manager was hired, there was some conflict between the medical assistant and receptionist, little sharing of information, and a lack of team decision-making. For example, Dr. Wagner wanted more patients steered to Dr. Turner while staff wanted patients directed away from him because it disrupted patient flow. As expected, early in the RAP meeting process one of the staff criticized the doctors for their different disruptive styles. However, by the fifth meeting, the RAP team was handling two to three issues every week. The doctors seemed to have become quite comfortable letting staff speak up and voice disagreement, and listened as staff members made suggestions. There were many conversations going on outside of RAP that were helping the work of the RAP team. Two years later, the practice was still having RAP meetings every two weeks and had expanded these to include the second site.

### Walker Family Medicine

Walker Family Medicine was a large practice with five full-time and one part-time physician occupying two floors of a professional building in suburbia. The long-time office manager (OM) closely directed all operational matters for the practice, and sought to maintain stability so doctors could focus only on patient care. S/he had a no-nonsense, command and control, directive style that rewarded staff according to her/his vision of the smoothly operating medical office. With suggestions from the ULTRA facilitator, the practice formed a RAP team consisting of the OM and key mid-level supervisors. The latter consisted of individuals closely connected with OM, but, unfortunately, also seen as "her/his favorites" among the large practice staff. The RAP team initiated constructive meeting conversations. While these sessions brought forth some new and helpful ideas, OM would often reframe issues to fit her/his agenda, and stifle the emergence of truly creative ideas inside and outside of RAP meetings. At the same time, however, a general distrust of the RAP team and "what they were up to" rippled through the practice leading to fear that RAP team activities might endanger some jobs. These unanticipated conversations became so disruptive that OM asked the facilitator to meet separately with the rest of the practice to address these fears and provide reassurance.

These three stories show how conversation in practices affected, in surprising ways, our intervention efforts. For instance, in Walker Family Medicine and Belton Clinic, our intervention did not progress as well as expected because unanticipated conversation emerged and blocked the intervention. Sometimes conversation changed the effect of our interventions for the better in ways we did not expect, as in Stanton Family Medicine where unanticipated conversations were generated and changed the relationship system, thereby facilitating the intervention. Even though our particular intervention involved discussion between a select set of clinic members within RAP meetings, informal conversation that took place outside of these meetings and among all clinic members greatly influenced this intervention.

In the next section of the paper, we note that complex adaptive systems theory is a useful framework for conceptualizing health care organizations. In particular, it causes us to focus on the role of relationships and to see the role of conversation in interventions. Utilizing concepts from sociolinguistics, we then clarify a definition of conversation, distinguishing it from notions such as instruction-giving and information-exchange. We articulate the role of two organizational actions important for intervention success, sensemaking, and learning. We explore ways in which conversation can enhance interventions by improving sensemaking and learning, and ways in which conversation can reduce intervention success by inhibiting sensemaking and learning. Finally, we suggest specific activities for stakeholders as they seek to understand and use conversation effectively as an important aspect of successful health care interventions. Throughout, we present observations from our own intervention studies.

## Discussion

### Health care organizations as complex adaptive systems

When health care organizations are seen as mechanistic systems then interventions are seen as strategies for fixing broken parts and putting them back correctly to improve system functioning. Unexpected variability in outcomes of intervention efforts is often attributed to incorrect execution of the intervention [1,6]. The prevailing assumption is that surprises in intervention outcomes can and should be avoided with more knowledge, or better intervention design, quality control, planning, and standardization [2,4]. Health care practices seen as complex adaptive systems have structures, processes, and functions that resemble living organisms more than they resemble machines. From a complex adaptive system point of view, variation in outcomes of interventions is to be expected because surprise is often due to the fundamental nature of these systems [6]. When health care organizations are seen as complex adaptive systems then local relationships and interdependencies among organizational members become paramount to intervention success because relationships are recognized as a primary source of system functioning. The relationships among agents lead to learning, sensemaking, improvisation, self-organization, and emergence, and these are among the key properties that define these systems [17-20].

Complex adaptive systems are constituted by nonlinear interdependencies within a network of diverse agents [6,21-23]. Rather than order and structure being solely imposed from top-down mandates, directed by blueprints or plans, or controlled by outside leaders or rules, order and structure also spontaneously come about through self-organization. In self-organization, the effects of local interactions between diverse and responsive agents are amplified through a system even when no agent has the intention to affect the system [19]. Self-organization among agents at lower system levels leads to the emergence of patterns and order at higher levels; these are called emergent properties [24]. Depending on the nature of the interactions, these emergent properties can reinforce existing patterns or create system change. Because multiple interactions among agents occur simultaneously and because agents reciprocally influence one another, the dynamics of a complex adaptive system are nonlinear and frequently unpredictable.

These characteristics of health care organizations as complex adaptive systems have ramifications for our attempts to intervene in their functioning. While traditional conceptions of interventions emphasize careful construction and crafting, complex adaptive systems theory begs that we broaden our conception of interventions beyond core actions and outcomes. We must consider dynamic patterns, interrelated processes and relationships, and be open to unintended as well as unpredicted consequences. Because complex adaptive systems theory recognizes the centrality of interdependency and connectivity, it suggests that we design interventions that attend to the quality of relationships within a health care organization and between an organization and its environment. An organization's capacity to use and manage relationships is therefore critical to how they are going to manage intervention initiatives. Relationships can not be handled simply by appropriate division of labor, specialization, etc., because health care organizations exist in a world where interdependencies within and between systems give rise to unforeseeable events [9,20,25-27]. Change in a complex adaptive system is affected not only by the new intervention efforts but also by the routines and procedures that already exist in an organization because complex adaptive systems exhibit path dependence. Whenever a new intervention is adopted, it changes the ability of the organization to adapt to subsequent interventions. Existing sets of routines and procedures interact nonlinearly to enhance some innovations and inhibit others. An intervention that works for one organization may not work for another [28]. Thus, intervention attempts are rarely, if ever, simple matters of high fidelity transfer. The work of organizational change, therefore, consists not of designing new structures [to transfer to any organization] but of introducing new themes into the organizational conversation in the hope that they will "amplify and disseminate" [10].

Rather than emphasize the rule-bound, fixed, established, and enduring nature of communication [29], complex adaptive systems theory leads us to see conversation as a phenomenon emerging from iterative reciprocal interactions among individuals. Rather than seeing conversation as a process of exchanging or transferring information from one individual to another, we see it as a combination of rule-following and situated adaptation done by interacting participants locally adjusting their actions to contingent circumstances [13]. Because these interactions are multiple, interdependent, and occurring simultaneously throughout an organization, the dynamics of conversation are nonlinear, as are the resulting patterns of meaning and relating that are so important in intervention success [10,30]. In addition to creating and maintaining cohesion, conversation can also facilitate disruption and change by creating opportunities for new properties to emerge in an organization. We saw this in Stanton Family Medicine where new conversation changed the organization from being typified by conflict among members, little sharing of information, and a lack of team decision-making to an organization typified by voicing disagreement, making suggestions, and handling important issues related to our intervention. Conversation that has gone bad can also block productive change, as we saw in Belton Clinic where the clinic manager used conversation to deflect practice problems to the hospital network, argue with the RAP facilitator, and disrupt the intervention.

### Defining conversation in health care organizations: what conversation is; what it is not

In order to understand how conversation affects interventions, it is helpful to carefully define conversation and explicate the mechanisms through which this happens. Although important observations and insights have been made by organizational communication theorists examining formal, planned communication structures [12,31], institutional effects on communication [29,32], rule-bound regularities and stable determinants of communication, relationship behavior [33], and individual's interactional responses to planned change[11], for the purposes of this article we draw mostly on sociolinguistic understandings of conversation.

Sociolinguistics is often applied to locally occurring verbal exchanges between small groups of individuals. Also, many sociolinguistic understandings of conversation are compatible with our conception of health care organizations as complex adaptive systems. Many sociolinguistic scholars focus on the "continuous and spontaneous pattern-making of moment-to-moment interaction" [10]. For example, Hymes explored how the speech situations, events, and acts are particular to a community and how these emerge from local interactions [34]. Erickson argued that conversational theories must try to account for the joint presence of stability and change in social patterns [13]. He critiqued functionalism for overemphasizing socialization and rule-following as an explanation for the existence of social order, saying regularities in social interaction are the result of social agents learning and acting in ever-changing environments without intention or full reflective awareness. He claimed that people do not follow rules so much as they use rules as they size up their situations and act from moment to moment.

Our reading of sociolinguistic literatures causes us to use a definition of conversation involving three concepts: collaboration, meaning-making, and improvisation. First, conversation is a social act of collaboration [16,35]. Spoken or written turns, or comments, are traded back and forth and each turn relates in some way to the turn before it. These verbal exchanges are often amplified and clarified through non-verbal signals such as facial expressions, hand gestures, and body posture. Because neither the sequence, allocation, or content of conversational turns are pre-specified, participants must make an implicit agreement to collaborate by trying to understand one another and to be understandable to others [13,35,36]. Rather than this "rule" being imposed from outside, this oft-evoked global pattern of relating is better thought of as a self-organized, emergent response to the unpredictability of conversational interaction.

Second, exchanges between participants lead to collectively generated ideas, the meaning of which arises in the interaction among turns [10,37]. Thus, rather than information being simply passed intentionally and without change, meaning is created as conversation is jointly constructed. In the language of complex adaptive systems theory, one might say that meaning emerges from the self-organization of diverse and responsive agents [10]. The meaning created through dialogue varies greatly in its novelty, ranging from the reinforcement of old beliefs or strengthening existing relationships and power structures to completely innovative ideas existing in the mind of neither individual prior to the conversation. Through conversation, focus of this meaning is narrowed or broadened and options are selected, clarified, reduced, added or created. Such meaning-making may be especially important during intervention attempts.

Third, understanding among individuals can not simply be assumed because conversation is not completely scripted, but is collectively improvised. Neither ritualized nor random, it falls somewhere in the middle [13]. Like all self-organization, conversation requires the simultaneous presence of order and disorder, constraint and freedom [10]. Individuals improvise on a situation, using a combination of rule-following and situated adaptation. Some aspects of conversation are predetermined, and have become predictable by historical usage and convention [38]. For example, in the standard medical history-taking sequence the physician inquires about symptoms, the patient responds, and the physician acknowledges and evaluates [39]. At the same time, every conversation is unique and unpredictable in its unfolding. Improvisation is a required conversational skill due to the ambiguous nature of language and discourse. While some sociolinguists emphasized the structured, predictable nature of discourse [40,41], Sawyer claimed that our overriding tendency to assign single, centralized control causes us to assume that conversation is more scripted than it is [42]. Individuals don't just follow conversational rules; they use them to size up their situations and act from moment to moment. Patterns of relating and meaning continuously emerge from infinite configurations of situations and participants locally adapting themselves to contingent conditions [13]. We argue that conversation for the purpose of generating or facilitating intervention efforts must have elements of adaptable, flexible improvisatory response.

We distinguish conversation from instruction-giving and information exchange in which ideas are passed around but not created; or speeches, in which talk time is monopolized and turn-taking is nonexistent. Talk that is unidirectional, with all turns allocated to one party, does not qualify as conversation because it is not jointly constructed. Such is often the case during large group meetings. Also, talk that elicits no real new meaning is not conversation. An example is the highly formulaic sequence of, "Good morning, how are you?" "I'm fine, how are you?" We participate in these rituals so often that we may take part in them without making new meaning from them. Thus, in our conceptualization these types of exchanges are not conversation. That is not to say that such exchanges are not important. For instance, they may be an important ritual for maintenance of the relationship system within an organization, and that relationship system can subsequently determine if new meaning is an emergent property of a future conversation.

Although our definition of conversation emphasizes the local nature of conversation, as does our reliance on complex adaptive system theory, the term "local" in this context should not be taken to mean necessarily local in space. Rather, local is meant simply to convey the fact that we are limiting our discussion to conversation among organizational members and excluding inter-organizational discourse and talk that goes on with outsiders. While it is tempting to equate conversation with face-to-face forms of communication, our definition of conversation includes written exchanges when they are characterized by the necessary conditions of collaboration, meaning-making, and improvisation. Increasingly, conversation is mediated through technology and occurs in written form, sometimes asynchronously and sometimes virtually [43,44]. In primary care practices, the emergence of electronic medical records offers opportunities for virtual conversations that may involve patients, physicians, and practice staff.

### The role of sensemaking and learning in intervention

Fidelity during adoption of change initiatives has historically been taken for granted, the assumption being that implementers would copy or imitate an innovation exactly. Adopters were considered passive receivers of interventions as they were designed, rather than active transformers of ideas and plans [45]. Perhaps one of the reasons we have so much trouble implementing interventions is that it is not a transfer problem as we often conceive it to be. There is no sense in bemoaning the lack of fidelity in implementing interventions as originally conceived because a linear mapping between original conception and implementation in any particular context is highly unlikely and thus should not be assumed. Instead of thinking of intervention implementation as a problem of reliable transfer, we would be better off to think of it as a problem of sensemaking and learning.

When health care organizations are seen as complex adaptive systems it becomes clear that sensemaking and learning play a critical role in intervention success [46]. Sensemaking and learning emerge from systems of relationships and are affected by both the quality and quantity of conversation in which organizational members engage [47]. In the following sections we define sensemaking and learning, paying attention to their role in interventions. We then identify qualities of conversation that are important to sensemaking and learning. We wish to acknowledge that people in groups will always make sense of the world they encounter and will always learn strategies for engaging with that world. However, one can make sense of the world in ways that are dysfunctional with respect to achieving his/her goals, and one can learn in ways that block him/her from achieving goals. That said, we argue that high quality conversation can increase the likelihood that health care organizations will make sense and learn in ways that enable them to achieve their goals and to serve their stakeholders, including patients and providers, in positive ways. In other words, we want to do better.

### Sensemaking

When organizations and organizational members encounter intervention initiatives, they are often encountering non-routine problems, difficult decisions, ambiguous and conflicting information, shifting goals, time pressure, and dynamic conditions. In such situations it is critical that people not act on autopilot or normalize change out of existence, as may be their tendency. Rather, organizations need the capacity to continually make sense of dynamic situations if they are to successfully respond to interventions. "Sensemaking is a diagnostic process directed at constructing plausible interpretations of ambiguous cues that are sufficient to sustain action" [48]. In the face of intervention initiatives, people have to make sense of the intervention and what it means for them and for their organization. Sensemaking is not just a crutch that human beings use because of our limited cognitive capacity; it is a highly adaptive response in the face of fundamental uncertainty of a complex dynamic world [46]. Sensemaking unfolds in a nonlinear fashion and is interactive and relational [48]. Opportunities for sensemaking occur daily in medical practices. When a nurse notices a doctor falling behind and that the front desk staff continues to add extra call-ins, the situation can quickly become senseless. Practice members need to stop and talk across systems, thereby creating an impromptu conversation. Sensemaking is enhanced when the nurse checks in with the physician, finds out how long she thinks she is going to take, then relays that information back to the front desk where a conversation ensues about how the practice can best manage the situation.

Sensemaking is "an issue of language, talk, and communication" [48]. Through conversation, people make sense of their collective circumstances and of the events that affect them, and they create the basis for action to deal with those circumstances and events [47]. Practice staff and clinicians may fully understand the specifics of an improvement effort, but it is through conversations that they produce a shared vision of how a given intervention will improve care of their patients and will enhance real adoption of a change. Through conversation, people organize their group thinking about a problem, jointly develop possibilities for coordinated action within and between systems, and check assumptions. These facilitate sensemaking that leads to action [48]. Accepting, implementing, leveraging, and maintaining core interventions require practice members to make sense of their changing situations. Such collective sensemaking may be accomplished through narrative storytelling used to interpret a surprising event. Sensemaking narratives tend toward the nonlinear, with multiple story tellers/creators contradicting and interrupting, offering justifications, presenting multiple possibilities, and delineating dilemmas [49]. Such conversations were typical in the evolution of the RAP team in Stanton Family Medicine where the physicians' willingness to let staff speak up and voice disagreement facilitated sensemaking through multi-voiced storytelling.

### Learning

In order for an intervention to be successful, a health care practice must modify its perceptions, beliefs, actions, and behaviors. In other words, the practice must learn. Language is the medium through which humans think, and conversations are the medium through which individuals think together [14,35] and through which organizations learn.

"In traditional views of organizational life, knowledge is the key but in a complexity view, learning is the key" [46]. Traditionally, interventions are conceptualized as specific activities, behaviors, or beliefs that are transferred from the heads of researchers, designers, and facilitators to members of a health care organization. This transfer is sometimes thought to be accomplished through faithful implementation of a carefully planned process also known *a priori*. Learning is thought to be an intentional and easily directed act. From a complex adaptive system perspective, learning must take place as the world continuously unfolds. It is not possible to first learn about an intervention, then plan the intervention, and then implement the plan. Rather, individuals and collectives must learn as they act and they must act in order to learn [25]. From a complex adaptive systems standpoint, learning must occur in the face of only partial knowledge because nonlinear interdependencies make identification of causal linkages and prediction impossible. Because intervention attempts in complex adaptive systems are dependent upon nonlinear interdependencies, their effects are also uncertain. Therefore, intervention facilitators should pay attention to learning as it is occurring and not assume that what was intended by the intervention as originally conceived will be what is learned.

When practice members converse with each other they learn about their own thoughts and ideas and they collectively generate new ideas. Successful adoption of change has been found to be associated with conversations and collective learning processes in health care teams [50] When things are stable, organizational members may be able to get by with more scripted dialogue in their daily talk. But when a health care organization is desirous of change then conversational improvisation is needed to facilitate learning, questioning of beliefs and practices, and building new knowledge. For example, when a nurse practitioner notices an error had been made with a patient, an opportunity for learning can be created. The nurse practitioner who quickly pulls together her/his clinical team to talk through how this happened, and how they can avoid it in the future, is helping to create a culture where learning is expected and valued. Unfortunately, learning is often inhibited in health care organizations by the ways that organizational members are socialized, and by existing routines and status relationships. Often, this is referred to as a competency trap [51]. Competency traps block conversation and decrease the likelihood of success of intervention initiatives. Thus, it is less important for change agents and other leaders to understand and tell others what to do than to create an organizational culture where learning is highly valued, and where people pay attention to and respect diverse insights and understandings [46,52]. Creating an environment in which learning is highly valued was part of the impetus for the use of RAP teams in ULTRA (see number seven in Table 1).

Complex adaptive systems theory helps us understand the uncertain nature of the dynamics that take place in health care practices, especially during intervention attempts. Things often unfold in ways that are surprising and in ways that require that special attention be paid to the activities of sensemaking and learning. It is through continued attention to sensemaking and learning that a practice can change in response to interventions in ways that are productive for the practice and all of its stakeholders. In a complex adaptive system, a one to one matching of the way people interpret an intervention and respond to that intervention is highly unlikely. Systems for sensemaking and learning, and in particular, conversation as a mechanism for sensemaking and learning, are critical if we want interventions to positively affect the life of a health care organization.

### Qualities of conversation that improve sensemaking and learning

Conversation that improves sensemaking and learning depends on diverse partners who trust each other; who are responsive in their interactions through empathetic listening, paying attention, questioning each other, suspending assumptions, and expecting and dealing with misunderstanding. Trust develops when participants know each other well enough to behave with sensitivity toward one another, and to pace the discussion appropriately. When these conditions are present, practice members can engage in intimate exchange through the display of emotions that establishes authenticity and mutual appreciation. They can participate in respectful, disciplined debate as a source of vigorous questioning ensuring that relevant information is available within a group, and they can take part in creative dialogue that is deeply grounded in facts, but also in hopes and aspirations [53]. Sensemaking and learning are enhanced under these conditions. In Stanton Family Medicine, the physicians' willingness to let staff speak up and voice disagreement and to listen as staff members made suggestions likely contributed to the success of the RAP intervention.

### Qualities of conversation that inhibit sensemaking and learning

Capacity for sensemaking and learning can be inhibited when there is not enough time or space for conversation. Members of health care organizations often get so rushed that conversation seems like a waste, particularly when we believe that everyone should know what they are doing. Such was the opinion of Dr. Smith in Belton Clinic, who felt that time and space allotted for clinic conversation detracted from generating revenue. Even with adequate time and space, capacity for sensemaking and learning can be diminished when participants fail to engage in empathetic listening, as listening is often the main behavior of people engaged in conversation.

People may fail to listen empathetically when they think they know what others will say, assume agreement, focus on themselves instead of focusing on a topic, or tune out because they don't perceive that they will get an opportunity to speak [54]. Additionally, too much agreement too quickly can shut down conversation, thus limiting conflict, respectful argumentation, and diversity of ideas needed to create and evaluate opportunities for change [55]. In one clinic, we heard about a clinic meeting in which someone complained about problems with the phone system. The office manager squelched the conversation by quickly reporting that the clinic had already fixed the phone system and had spent a lot of money doing it. There was no more discussion.

Patterns of interaction within health care clinics tend to become routinized in systematically-organized ways of talking called discourses[38]. Dominant discourses may facilitate sensemaking and learning in that they can give expression to the meanings and values of an organization, and help to establish and maintain group identity and social integration. But dominant discourses can also create barriers to sensemaking and learning by their propensity to colonize and overpower diverse ways of thinking, acting, and conversing; and thereby decreasing flexibility, adaptability, and the ability of organizations to change [38,56]. The situation seems to be exacerbated in health care organizations, which tend to be siloed by specialty, each with their own dominant discourses between which few ties are forged [57,58]. This may have been operating in Walker Family Medicine where the RAP team consisted mostly of mid-level supervisors affiliated with a controlling office manager.

### Recommendations for enhancing the role of conversation in improving interventions

When one attempts an intervention, organizational members may already be conversing in ways that improve sensemaking and learning, or they may be conversing in ways that inhibit sensemaking and learning. The success of the intervention is affected by conversations that are taking place. Whether conversation existed prior to the intervention or comes about during the intervention, change agents can influence the qualities of conversations that make a difference to intervention efforts. We suggest six strategies that can enable conversation to improve rather than inhibit the sensemaking and learning needed for intervention success.

### Evaluate existing conversation and relationship systems

Conversation is an ongoing aspect of organizational life that continuously shapes the way members perceive their environment, their patients, and their tasks. Preexisting relationships can be a barrier or a facilitator of intervention attempts. Intervention change agents must determine to what extent these relationship systems are likely to encourage productive conversation. They should not overestimate their ability to predict the conversational potential of a practice, and instead continually observe, assess, and evaluate [59]. When relationships are strong and conversation is thriving, these should be leveraged to support an intervention.

One consistent finding across our own intervention attempts is how little people in health care practices talk about things that are relevant to the practice. Intervention leaders may well find health care situations where there are almost no conversations. Time pressures in health care life lead to situations where everyone goes from task to task, never having time to talk. Change agents may easily identify issues that need to be addressed, but unable to get a conversation going because everybody is so busy that there is no space within which to converse.

Potential for conversation is highly influenced by personal relationships, and these need to be evaluated on an ongoing basis. Particularly close relationships, such as family or family-like relationships can curtail members' ability to address issues that affect their organization. For example, when the office manager is the spouse of the lead physician, staff may have difficulties talking to each other or to either of them about the problem of the lead physician having difficulty keeping within the allotted time for appointments.

### Look for and leverage unexpected conversation

Complex adaptive systems theory suggests that existing conversations will take unexpected directions and change agents need to capitalize on the positive potential of unexpected conversations, and manage potentially negative conversations that they did not predict and can not control. They should be on the lookout for how conversation *is *changing during an intervention and how conversation potentially could change, given that the relationships in the practice and the intervention are unfolding together. They should also be open to the unique circumstances of fortuitous happenings that occur as conversations are collectively improvised [13]. For instance, in Walker Family Medicine the RAP facilitator was able to manage the unexpected conversations occurring outside of RAP meetings that may have undermined the intervention.

In one health care setting, when a new patient arrived staff realized that a wheelchair was needed for transportation due to his mobility issues. Staff was unable to find a wheelchair because two other patients had been sent to the emergency room that morning and the wheelchairs hadn't come back yet. Members of the clinical staff joined the front desk staff in organizing themselves to improvise a wheelchair out of swivel chairs. The office manager brought the staff together at the end of the morning, before people got away, to discuss how the practice could improve the way they managed these types of situations. By doing so, she capitalized on all the little conversations that had gone on around the wheelchair incident that morning to address a more global aspect of the clinic's functioning.

### Create time and space where conversation can unfold

Many health care organizations feel that creating time for conversation is not practical in their hectic environments. Nonetheless, rich conversation is a critical part of adapting an intervention and making it successful. Intervention leaders should integrate structural elements into intervention efforts to help people have informal conversations about an intervention. Such conversations can enable the sensemaking and learning needed for an intervention to be successful. For instance, after formal training sessions for the use of an intervention, time can be allotted for informal conversations. This can often be implemented by such things as refreshments and coffee hours. Sharing a boxed lunch before a training session on the use of a new technology can provide an informal setting for the expression of anxieties about the upcoming program.

Given the dynamic, recursive, and iterative nature of change, intervention agents must also protect time and space for conversation to unfold. Stanton Family Medicine was diligent in protecting time for conversation. Not only did practice members (with the encouragement of the facilitator) conscientiously meet for discussion during the intervention, the clinic was still consistently protecting time at three-year follow-up. As a clinic member said, "meetings take time from the doctor's schedule, but they are an important function of this office. I don't see us not having these meetings."

### Use conversation to help people manage uncertainty

Providing opportunities for organizational members to freely voice their nervousness and their excitement about change efforts can help people prepare for, make sense of, learn about, and reflect on the uncertainty that change often creates. Uncertainty is a constant feature of the health care landscape and will be exacerbated by serious intervention efforts. Significant change often requires interruption of established discourses and conversational patterns, as well as modification of perceptions, beliefs, actions, behaviors, or even identities [50]. We often do not recognize how much stress intervention attempts cause as people try to manage performance concerns, normative concerns, and uncertainty concerns. When there is a lot going on, people instinctively get together and talk about things, and these interactive coping tactics can be benign, neutral, or destructive [11]. For example, changing reporting relationships in a work group may improve effectiveness and efficiency but it will certainly reorder personal relationships, and this will certainly cause stress.

### Use conversation to help reorganize relationships

Because relationships are critical to intervention success, using conversations to reshape relationships is a significant strategy for intervention leaders. Intervention leaders should create ways for people to talk to one another who normally do not talk. In our recent research, the RAP process in Stanton Family Medicine began with selected participants from the initial practice, and evolved to integrate participants from a second site into a single set of conversations. Intervention leaders can also generate tactics to help people talk together in new ways, for instance by changing the frequency of their interaction, their topics of discussion, and the ways in which conversation unfolds. In a recent study of difficulties in adopting new cardiac surgical techniques, Edmondson discovered that bringing people together to learn the new technique can reframe relationships among the members of a cardiac surgical team [50]. When introducing a new technology, one can encourage the conversation around this intervention to extend to cover the entire care of the patient, instead of focusing exclusively on the new technology.

#### Enhance conversational capacity by building social interaction competence

Acknowledging that conversation is a critical component of all interventions, change agents should help people associated with an intervention pay more attention to conversation and developing social competence [60]. It is important to encourage and help organizational members seek feedback about their conversational efforts, and to teach them to utilize strategies that might enhance conversation, such as inviting respectful argumentation, disciplined debate, creative dialogue, and intimate exchange [53]. Change agents should facilitate people's understanding of conversational barriers so that they might develop strategies for tearing them down; for instance, by inviting diversity and engaging in empathetic listening. In Stanton Family Medicine, the intervention facilitator encouraged the physicians to respond positively to staffs' criticisms, disagreement, and suggestions, which enabled the RAP team to explore new ideas that might otherwise have been stifled as was the case in Belton Clinic.

Formal conferences and huddles are two occasions where opportunities for building social interaction competence can be easily overlooked. Formal conferences are one of the few places where physicians practice talking to each other about difficult clinical issues. Social interaction skills learned in the context of formal conferences are more likely to transfer to physicians' own settings when a conscious effort is made to facilitate that transfer.

Huddles are short daily meetings focused on adjusting to the day's idiosyncrasies, such as missing organizational members/being short-staffed, challenging patients scheduled back-to-back, last-minute scheduling changes, and equipment failures. Huddles address immediate coordination issues and offer opportunities to develop a different set of social interaction skills. However, because the huddle looks so simple, people often do not pay enough attention to the development of these skills. Intervention leaders, using huddles, can help organizational members learn to focus attention quickly, participate in the conversation irrespective of status or rank, pay special attention to listening to others, avoid bring extraneous issues into conversations, and leave conversations with specific action objectives. The "on-the-go" nature of huddles increases their potential for transfer to more improvisatory, informal conversation. [61].

## Summary

The theory developed here is grounded in both the literature of complex adaptive systems theory and of sociolinguistics and supported through empirical observation of primary care practices studied as part of a fourteen-year research program. The role of communication and conversation in intervention has long been a concern to communication scholars [11,12]. We add to this literature by developing a theory that shows how conversation can affect the sensemaking and learning necessary for successful interventions in health care organizations.

Health care organizations, because they are complex adaptive systems, are fueled by conversation that constitutes relationships. If we are attempting to enhance the way that cardiac surgical teams learn new procedures, help hospitals develop new patient safety protocols, or help nursing homes provide more sensitive care to residents, then we need to recognize that the conversation among stakeholders will be critical to the success of our efforts. There are several key take home lessons that transfer across intervention efforts and across many different kinds of health care organizations. Table 2 summarizes our key points.

**Table 2:** Conversation summarized

**Definition of conversation**	**Emphasize qualities of conversation that improve sensemaking and learning**	**Avoid conversation that inhibits sensemaking and learning**	**Recommendations for enhancing the role of conversation in improving interventions**
What it is • Collaboration • Meaning making • Improvisation What it is not • Instruction-giving • Information exchange • Speeches • Talk that elicits no real meaning	• Trust • Responsive interaction • Empathetic listening • Diversity of perspectives • Intimate exchange • Disciplined debate • Creative dialogue	• Lack of time and space • Failure to listen • Too much agreement • Dominant discourses diminish diverse perspectives • Siloed specialties	• Evaluate the potential of an intervention to generate conversation • Look for and leverage unexpected conversation • Create space within which conversation can unfold • Use conversation to help people manage uncertainty • Use conversation to help reorganize relationships • Build social interaction competence

Conversation is really hard. It is easy to say, "the issue is communication," and it is easy to say "we have to talk to each other." But it is hard to collaborate, make meaning, and improvise. It is difficult to create conversation that facilitates sensemaking and learning, and avoid the barriers to conversation that facilitates sensemaking and learning. Even though conversation is hard, we really have to do it if we want to deliver good health care.

Conversation may be particularly challenging in health care organizations because of information asymmetries and the need for confidentiality, among other things. Within health care organizations, sensemaking and learning are critical and often overlooked, and conversation is essential for effective sensemaking and learning to occur. When health care organizations are seen as complex adaptive systems and conversation is viewed through a sociolinguistic perspective, then conversation is recognized as a critical mechanism through which self-organization occurs and by which patterns of relationship are created. If we want to intervene in the way health care organizations do business, then we must pay attention to the role of conversation in intervention outcomes.

## Competing interests

The authors declare that they have no competing interests.

## Authors' contributions

MJ led the writing of the paper to which all authors participated in providing critical input. MJ and HL developed the conceptual frameworks and the basic theoretical arguments. BC and KS were principle investigators on the studies from which the ideas in this paper emerged and also provided input into the role of conversation in the studies they conducted. PN and WM contributed to the design of the manuscript and in analyzing the role of conversation in practice change efforts. PN, WM, and BC reviewed the primary data and constructed the case examples. RM provided conception and design input. All authors reviewed the manuscript at all stages of its development; all authors read and approved the final version.
